# Evaluation of sexual history-based screening of anatomic sites for chlamydia trachomatis and neisseria gonorrhoeae infection in men having sex with men in routine practice

**DOI:** 10.1186/1471-2334-11-203

**Published:** 2011-07-26

**Authors:** Remco PH Peters, Stephan P Verweij, Noëmi Nijsten, Sander Ouburg, Johan Mutsaers, Casper L Jansen, A Petra van Leeuwen, Servaas A Morré

**Affiliations:** 1 The Hague Municipal Health Services, STI clinic, The Hague, The Netherlands; 2 ANOVA Health Institute, Khutšo Kurhula Offices, Tzaneen, South Africa; 3 Laboratory of Immunogenetics, Department of Pathology, VU University Medical Center, Amsterdam, The Netherlands; 4 Department of Medical Microbiology, MCH Westeinde Hospital, The Hague, The Netherlands; 5 Cluster of Infectious Diseases, Public Health Service Amsterdam, Amsterdam, The Netherlands

**Keywords:** Chlamydia trachomatis, Neisseria gonorrhoeae, anorectal, oropharyngeal, screening

## Abstract

**Background:**

Sexually transmitted infection (STI) screening programmes are implemented in many countries to decrease burden of STI and to improve sexual health. Screening for *Chlamydia trachomatis *and *Neisseria gonorrhoeae *has a prominent role in these protocols. Most of the screening programmes concerning men having sex with men (MSM) are based on opportunistic urethral testing. In The Netherlands, a history-based approach is used. The aim of this study is to evaluate the protocol of screening anatomic sites for *C. trachomatis *and *N. gonorrhoeae *infection based on sexual history in MSM in routine practice in The Netherlands.

**Methods:**

All MSM visiting the clinic for STI in The Hague are routinely asked about their sexual practice during consulting. As per protocol, tests for urogenital, oropharyngeal and anorectal infection are obtained based on reported site(s) of sexual contact. All consultations are entered into a database as part of the national STI monitoring system. Data of an 18 months period were retrieved from this database and analysed.

**Results:**

A total of 1455 consultations in MSM were registered during the study period. The prevalence of *C. trachomatis *and *N. gonorrhoeae *per anatomic site was: urethral infection 4.0% respectively and 2.8%, oropharynx 1.5% and 4.2%, and anorectum 8.2% and 6.0%. The majority of chlamydia cases (72%) involved a single anatomic site, which was especially manifest for anorectal infections (79%), while 42% of gonorrhoea cases were single site. Twenty-six percent of MSM with anorectal chlamydia and 17% with anorectal gonorrhoea reported symptoms of proctitis; none of the oropharyngeal infections were symptomatic. Most cases of anorectal infection (83%) and oropharyngeal infection (100%) would have remained undiagnosed with a symptom-based protocol.

**Conclusions:**

The current strategy of sexual-history based screening of multiple anatomic sites for chlamydia and gonorrhoea in MSM is a useful and valid guideline which is to be preferred over a symptom-based screening protocol.

## Background

Many countries have implemented national programmes for screening for sexually transmitted infections (STI). The aims of such programmes are to decrease the general burden of STI, to improve physical and sexual health, and to reduce transmission and acquisition of STI. Screening for chlamydia and gonorrhoea has a prominent place in these programmes and is done opportunistically, *i.e*. regardless of the presence of symptoms, because most of the cases are asymptomatic and there is a low diagnostic accuracy of selective screening criteria [1]. The screening of high-risk populations for urogenital infection has been shown to be feasible and is associated with clear reductions in the incidence of STI [2-6].

The core component of screening for *Chlamydia trachomatis *and *Neisseria gonorrhoeae *infection in MSM is testing for urogenital infection through urine sample or urethral swab. During the past decade, various studies from the United States of America (USA) and Australia have highlighted the importance of testing oropharyngeal and anorectal samples in addition to urogenital tests in MSM [7-14]. For example, a study in San Francisco showed that 53% of *C. trachomatis *and 64% of *N. gonorrhoeae *infections in MSM involved nonurethral sites and would be missed if screening was done only for urethral infection [7].

The present United Kingdom National Screening and Testing Guidelines, Norwegian HIV and STI screening protocols, Australian STI screening protocols, and guidelines from the Center for Disease Control and Prevention include specific recommendations for screening for anorectal and oropharyngeal infection in MSM based on reported site of sexual contact [4-6,15-17]. The Dutch Society for Dermatology and Venereology has similar guidelines for screening for chlamydia and gonorrhoea in MSM: a urethral or urine sample should be tested together with an oropharyngeal sample in case fellatio is reported and an anorectal sample if passive anorectal intercourse is reported. Anorectal infections in MSM have special attention due to the identification of the lymphogranuloma venereum strain L2b among MSM [18,19]. These tests should be obtained regardless of the presence of symptoms. In contrast to the UK and CDC guidelines, testing for oropharyngeal *C. trachomatis *infection is included in the Dutch guidelines as optional. Data on the prevalence of oropharyngeal chlamydia in MSM is limited, but in most studies the prevalence is < 2% [7,20-22].

At the STI clinic in The Hague the guidelines of the Dutch Society of Dermatology and Venereology are used for screening for chlamydia and gonorrhoea in MSM. These guidelines include anatomic site specific testing based on sexual history, including tests for oropharyngeal *C. trachomatis *infection. While anatomic site-specific STI testing in routine care has been evaluated previously in some countries, only small studies have been undertaken in Europe [7-14]. In addition, to our knowledge only two studies have been conducted in a European setting to assess the value of this approach, but these included a relatively small number of MSM (39 and 599, respectively) [21,22]. In the present study, we evaluate the protocol of screening multiple anatomic sites for chlamydia and gonorrhoea in a large cohort of MSM at an STI clinic in the Netherlands to assess the usefulness of this screening strategy in routine practice.

## Methods

### Study population

The STI Clinic in The Hague, The Netherlands offers free and anonymous STI screening to people living in the city of The Hague and the surrounding region. Approximately 15% of visitors to the clinic are MSM. During routine consultation, sexual history is obtained using a standard questionnaire. This questionnaire includes questions about STI history, sexual practice, number of sex partners, site(s) of sexual contact, and symptoms that may be associated with STI. After counselling, clients are routinely tested for chlamydia, gonorrhoea, syphilis, hepatitis B virus and HIV. The local medical ethical committee approved this study, based on the fact that in the Netherlands ethical approval is not required for a retrospective, de-identified study.

### Screening protocol

The following protocol is used routinely for screening MSM for *C. trachomatis *and *N. gonorrhoeae *infection. First-void urine is collected from all asymptomatic visitors; a urethral swab is taken in case of penile discharge or dysuria. If fellatio is reported in sexual history, an oropharyngeal swab is obtained by wiping the swab twice over the lateral posterior sides of the pharyngeal wall followed by wiping once across the oropharyngeal wall. If receptive anal intercourse is reported, an anorectal sample is obtained by introducing a swab approximately 5 cm into the rectum under rotating movements. The swaps were clinician-collected without the use of a proctoscope.

### Data collection

All consultations at the clinic are recorded in an anonymous electronic file. This record includes basic demographic data, the routine questionnaire, results of microbiological tests, diagnosis and treatment if applicable. After completion of the episode, all records are transferred anonymously into a national database (SOAP) for surveillance purposes. In this study, we retrieved and retrospectively analysed data from the SOAP database for the STI clinic in The Hague over an 18 month period (January 2007-July 2008).

### Clinical definitions

We define urethral gonorrhoea or chlamydia as a positive test result for *C. trachomatis *or *N. gonorrhoeae *for a urethral swab, or urine sample by PCR. Urethral infection was considered symptomatic in case of dysuria or penile discharge. Pharyngeal chlamydia or gonorrhoea was considered symptomatic in case of a sore throat and rectal infection was defined symptomatic if anorectal discharge, blood or mucus in the stools, tenesmus or rectal pain was reported by the client.

### Laboratory tests

Specific urethral, oropharyngeal and anorectal swabs were used for specimen collection (APTIMA^®^; Gen-Probe Incorporated, San Diego, CA). Samples from each anatomical site were collected in separate sample collection tubes. Testing of samples for *C. trachomatis *and *N. gonorrhoeae *was done using the APTIMA Combo 2^® ^(AC2) assay (Gen-Probe Incorporated, San Diego, CA) according to the manufacturer's instructions. Positive reactions for *C. trachomatis *and *N. gonorrhoeae *with the AC2 assay were confirmed with the APTIMA CT (ACT) the APTIMA GC (ACG) assay respectively. Genotyping for specific detection of the lymphogranuloma venereum (LGV) strain was done on all anorectal samples with a positive result for *C. trachomatis*[23].

### Statistical analysis

The SOAP database was evaluated for missing, incomplete and conflicting data and where possible cleaned by reviewing free text in the records. Data were analyzed using SPSS version 13.0 (SPSS Inc., Chicago, IL) and described as numbers (%), proportion or median (range). Categorical data were compared between groups using Chi-square test and Fisher's Exact test when appropriate for categorical data and the Mann-Whitney test for continuous data. Risk factors were described as Odds ratio (OR) with 95% confidence interval (CI). The distribution of prevalence of chlamydia and gonorrhoea per anatomic site was depicted stratified by 5-year age groups.

## Results

### Characteristics of study population

During the study period a total of 1455 consultations by MSM were registered with complete data. This includes three transgender men who registered as women, but reported sexual contact with men and tests for male STI screening were obtained. The median age was 38 years old. The average sexual partners in the previous six months was four; 29 were commercial sex workers; 358 consultations had history of STI, and 128 were notified by a sexual partner. Clinical characteristics of the study population are summarised in table 1.

### Prevalence of chlamydia and gonorrhoea per anatomic site

Based on sexual history, tests for urethral infection were done in all except 4 consultations (99.7%); 1283 (88%) MSM reported fellatio and had oropharyngeal swabs taken while anorectal swabs were obtained from the MSM who reported passive anorectal intercourse (n = 1141; 78%). In the majority of consultations (n = 1095) samples were obtained from all three anatomic sites (75%), while 16% had two sites tested (n = 230) and 130 (8.9%) had only tests done for urethral infection.

**Figure 1:**
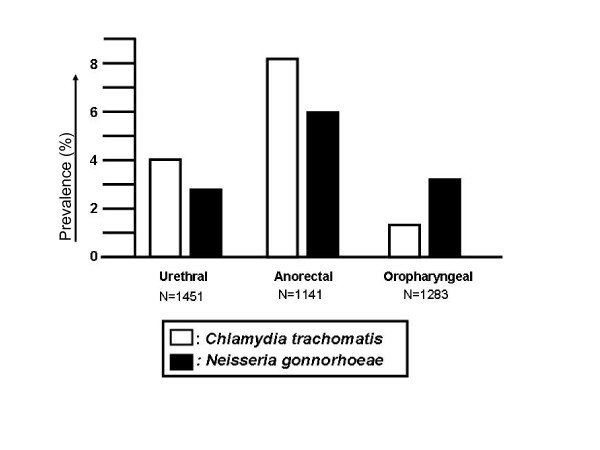
**Prevalence of Chlamydia trachomatis and Neisseria gonorrhoea infection per anatomic site in MSM**. MSM, men who have sex with men.

The overall prevalence of chlamydia was 10% and prevalence of gonorrhoea was 7.7%. The prevalence of *C. trachomatis *at the individual anatomic sites was: urethra 4.0%, oropharynx 1.5% and anorectum 8.2%, and for *N. gonorrhoeae *respectively 2.8%, 4.2% and 6.0% (Figure 1). Rectal *C. trachomatis *infection was diagnosed in 94 cases and the LGV strain was found in 5 (3.1%); all of these presented with symptoms of proctitis.

The majority of *C. trachomatis *infections (72%) involved a single anatomic site in those MSM with two or three anatomic sites tested. This was especially manifest for anorectal infection where 79% of cases were limited to the anorectum. Sixty-five percent of cases of urethral chlamydia and 53% of those with oropharyngeal chlamydia were single site infections. In contrast, the majority of *N. gonorrhoeae *cases (59%) involved multiple anatomic sites: only 26% of urethral, 43% of anorectal and 50% of oropharyngeal infections were limited to that specific anatomic site.

### Clinical presentation and risk factors

Symptoms were reported in the minority of consultations: 16% urethral, 4.5% anal and 1.4% pharyngeal, while the majority of cases of chlamydia (56%) and gonorrhoea (43%) did report symptoms. Predictive values of specific STI symptoms for chlamydia and gonorrhoea were low: urethritis was reported by 228 men (16%), but chlamydia was only diagnosed in 38 (17%) and gonorrhoea in 34 (15%). *C. trachomatis *infection was found in only 17 (26%) and *N. gonorrhoeae *in 11 (17%) of 65 individuals presenting with symptoms of proctitis. None of the cases with oropharyngeal chlamydia (n = 19) or gonorrhoea (n = 54) were symptomatic, but symptoms of pharyngitis were reported by 20 MSM without chlamydia or gonorrhoea. As such, if a symptom-based protocol would have been used for screening of anorectum and oropharynx, 121/145 (83%) of cases with anorectal infection and 68/68 (100%) of those with oropharyngeal infection would have remained undiagnosed.

**Figure 2:**
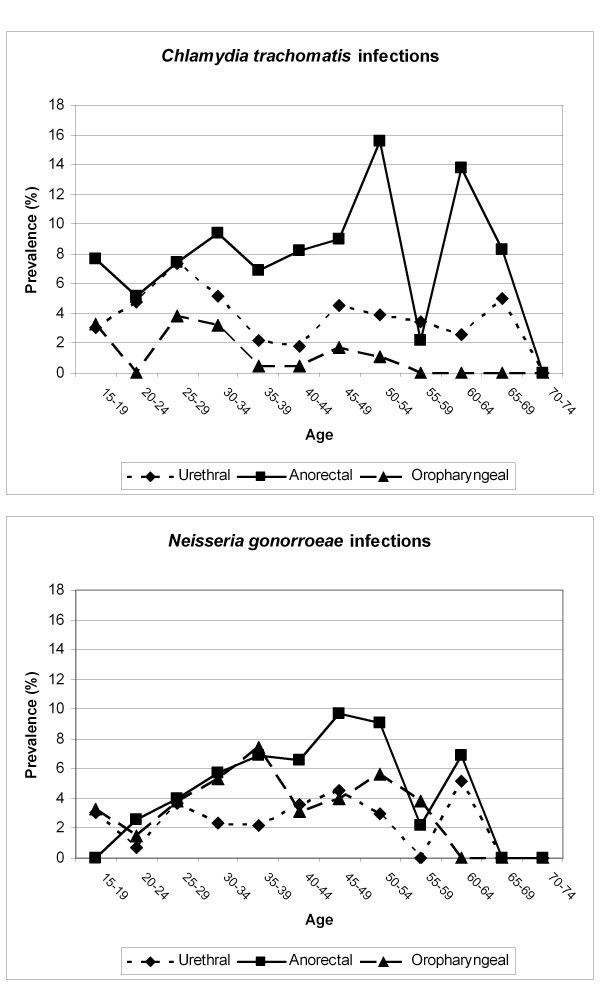
**Prevalence of *Chlamydia trachomatis *and *Neisseria gonorrhoeae *infection per anatomic site stratified by age groups**.

Median age of MSM with anorectal chlamydia or gonorrhoea was significantly higher than of those without anorectal infection (40 *vs*. 37 years; p = 0.02); this association was not observed for urethral (p = 0.6) or oropharyngeal (p = 0.4) infection. Figure 2 shows the prevalence of *C. trachomatis *and *N. gonorrhoeae *per anatomic site stratified by age group. The figure shows that the prevalence of anorectal infections is highest in the older age-groups whereas detection of urethral infections remains stable throughout.

The reported number of sexual partners in the period of 6 months prior to consultation was higher by MSM with chlamydia (mean 12 *vs*. 8.2; p = 0.03) or gonorrhoea (11 *vs*. 8.4; p = 0.03) than those without infection. This was especially related to anorectal infection (13 *vs*. 8.6; p = 0.02) but not the case for urethral (p = 0.5) and oropharyngeal infection (p = 0.09). In contrast, a history of STI was a risk factor for chlamydia or gonorrhoea infection at each anatomic site: urethra (OR 1.7 (95% 1.1-2.7); p = 0.02), oropharynx (OR 2.0 (95% CI 1.2-3.3); p < 0.01), and anorectum (OR 2.2 (95% CI 1.5-2.1); p < 0.001). In those with known HIV status (n = 1206), HIV infection was associated with increased risk for chlamydia (OR 2.5 (95% CI 1.6-4.0); p < 0.001) and gonorrhoea (OR 3.6 (95% CI, 2.2-6.0); p < 0.001). HIV seropositive status was especially associated with anorectal infection (OR 3.6 (95% CI 2.2-5.6); p < 0.001), to a lesser extent with urethral infection (OR 1.9 (95% CI 1.0-3.5); p = 0.04) and not with oropharyngeal infection (p = 0.1).

## Discussion

This study shows that testing of multiple anatomic sites for chlamydia and gonorrhoea based on sexual history is a useful strategy for screening for chlamydia and gonorrhoea in MSM. Our data support previous reports and national guidelines that suggest benefit of testing swabs obtained from oropharynx and anorectum in addition to urethral samples in MSM [4-14,21,22].

The prevalence of *C. trachomatis *and *N. gonorrhoeae *infection of oropharynx and anorectum in our study is in line with data reported by others [7-14,21,22]. Similar to those studies, we also found that urethral infections only represent a minority of cases and that anorectal infection is more common. In our setting, the prevalence of chlamydia and gonorrhoea more than doubled with testing of multiple anatomic sites compared to obtained tests for urethral infection alone. The majority of *C. trachomatis *infections involved a single anatomic site, which was especially the case for anorectal chlamydia, while only a small majority of *N. gonorrhoea *infections involved multiple sites. Altogether, our data strongly support the current guidelines that suggest screening anorectum and oropharyngeal samples based on sexual history in addition to urethral tests [4-6,15,16,18].

The prevalence of *C. trachomatis *in oropharyngeal samples was 1.5% which was in similar range as reported for a cohort of women reporting fellatio in the same setting [24]. Although there is sufficient evidence to screen for oropharyngeal gonorrhoea, there is ongoing debate about the relevance of screening for oropharyngeal Chlamydia [25-28]. This debate is about the prevalence and transmissibility of oropharyngeal *C. trachomatis *infection. The exact risk of transmission of *C. trachomatis *from throat to penis in fellatio is unknown, but a recent study suggests this may be quite considerable [29]. Altogether, in the absence of clear data about risk of transmission, it seems reasonable to include tests for oropharyngeal chlamydia in the routine screening protocol. Oropharyngeal swabs could be tested simultaneously for *N. gonorrhoeae *and *C. trachomatis *if a nuclear amplification test is used. In that regard, cost-effectiveness analyses are warranted; a possible method to reduce costs would be to combine swabs from different anatomic sites in a single collection tube and to test these samples simultaneously in a single reaction [30].

The current protocol of opportunistic screening of anatomic sites based on sexual history has a much better performance than theoretically would have been obtained with a screening strategy based on reported symptoms. The vast majority of *C. trachomatis *and *N. gonorrhoeae *infections were asymptomatic, regardless of the anatomic site involved. In addition, the positive predictive values of proctitis and pharyngitis are very low. As such, opportunistic screening is superior to symptom-based screening. We used reported sexual exposure as indication for obtaining anatomic site specific tests. This approach is in line with the current guidelines, but the reliability of sexual history in this context is unclear. Some men may not report exposure for reasons of stigma or embarrassment. An Australian study [31] states that approximately half of the anorectal infections were self-reported, where half were diagnosed by opportunistic screening. Thus an alternative strategy is opportunistic screening of all anatomic sites in all MSM regardless of reported exposure. Considering that the vast majority of our clients reported sexual contact at all three anatomic sites, that MSM can be very outspoken about their sexual practice (*e.g*. deny ever engaging in receptive anorectal intercourse), that the expected prevalence of infection at anatomic sites that were not exposed during sexual contact is low, and the physical burden of obtaining tests from patients denying sexual contact at that specific body site, we believe that opportunistic screening of multiple anatomic sites in all MSM is unlikely of additional value to sexual-history based screening protocol. Nevertheless, studies are warranted to confirm this hypothesis.

In this retrospective analysis we found that higher number of sexual partners, history of STI, and HIV seropositive status were risk factors for chlamydia and gonorrhoea infection at any anatomic site. These are known risk factors for STI and markers of high risk sexual behaviour. Risk factors associated with anorectal infection were older age, higher number of sexual partners, and HIV seropositive status. Our results show an increasing prevalence of both anorectal chlamydia and gonorrhoea infections with increasing age. As such, specific attention should be given to preventive measures and anorectal screening in MSM with those characteristics.

This study has several limitations. First, one client can be included in the database with multiple visits related to different consultations due to the set-up and anonymous character of the database. Based on some variables (age, postal code, and ethnicity), we estimate that 90% of the consultations are unique and 10% are multiple visits. These multiple visits may have introduced some bias when calculating risk factors for chlamydia and gonorrhoea at specific anatomical sites, because those are related to risk behaviour. Secondly, the data presented were collected during routine clinical consultations and only information included in the standard questionnaire was captured systematically. For example, the report of pharyngitis was only recorded if the patient complained about a sore throat, but not specifically asked for. As such, more studies are warranted to confirm our findings.

## Conclusions

Our data indicate that the current strategy of sexual-history based screening of multiple anatomic sites in MSM is a useful and valid guideline for screening for chlamydia and gonorrhoea in MSM and that this approach is superior to a symptom-based screening protocol. Specific attention should be given during consultation to information, prevention and anorectal screening of older MSM, those with a relatively high number of sexual partners, and those with HIV seropositive status.

## Competing interests

The authors declare that they have no competing interests.

## Authors' contributions

RPHP: study design, data/statistical analyses, drafting the manuscript

SPV: data/statistical analyses, drafting the manuscript

NN: data collection, critically reading manuscript

SO: data/statistical analyses, drafting the manuscript

JM: data collection, critically reading manuscript

CLJ: data collection, critically reading manuscript

APL: study design and conception, critically reading manuscript

SAM: study design, conception and coordination, critically revising the manuscript

*All authors contributed to writing of the final manuscript*.

All authors read and approved the final manuscript

## Pre-publication history

The pre-publication history for this paper can be accessed here:

http://www.biomedcentral.com/1471-2334/11/203/prepub
